# Corneal endothelial cell density and morphology in ophthalmologically healthy young individuals in Japan: An observational study of 16842 eyes

**DOI:** 10.1038/s41598-021-97776-5

**Published:** 2021-09-14

**Authors:** Takashi Ono, Yosai Mori, Ryohei Nejima, Takuya Iwasaki, Takashi Miyai, Kazunori Miyata

**Affiliations:** 1 Department of Ophthalmology, Miyata Eye Hospital, 6-3, Kuraharacho, Miyakonojo, Miyazaki 885-0051 Japan; 2 Department of Ophthalmology, Graduate School of Medicine, The University of Tokyo, Tokyo, Japan

**Keywords:** Diseases, Eye diseases, Anatomy

## Abstract

We aimed to investigate the cell density and morphology of the corneal endothelium in ophthalmologically healthy young Japanese, given the lack of normative data in literature. This observational study included eyes without ophthalmologic diseases, besides refractive errors, examined between 1996 and 2015 at Miyata Eye Hospital. Eyes with a history of ophthalmologic diseases or contact lens usage were excluded. Correlation of corneal endothelial cell density (ECD), coefficient of variation (CV), appearance rate of hexagonal cells (6A), and cell area with age were examined. Multivariate linear regression analysis was performed to determine the predictors of corneal parameters. We included 16842 eyes of 8421 individuals (19.6 ± 8.7 years). ECD was 3109.0 ± 303.7 cells/mm^2^ and significantly reduced with age (p < 0.001). The ECD reduction rate was 0.42%/year in the total population. On multivariate analysis, age and sex were significantly correlated with ECD, CV, 6A, and cell area (all p < 0.001). ECD, 6A, CV, and cell area are significantly associated with age in healthy young Japanese individuals. Monitoring their corneal endothelium is essential to assess the risk of endothelial damage.

## Introduction

Corneal endothelial cells constitute a single cell layer and help maintain corneal transparency by the barrier function that could let water and nutrients into the corneal stroma from the anterior chamber and the pumping function performed by Na–K-ATPase, thereby maintaining visual acuity^1^. Corneal endothelial cells can be damaged by various reasons, such as ophthalmologic surgery^2,3^, trauma^4^, uveitis^5^, contact lens usage^6^, ultraviolet radiation^7^, and aging^8^. Moreover, decreased corneal endothelial cell density (ECD) owing to the reasons mentioned above results in blurred vision or impaired visual acuity, which requires corneal transplantation for function recovery^9^. Therefore, it is clinically essential to observe the status of corneal endothelial cells.

Although it is necessary to determine its normal range in healthy patients to understand the degree of corneal endothelial cell damage, ECD or corneal endothelial morphology is known to vary across different populations, such as 2610 ± 372 cells/mm^2^ for Nigerian^10^, 2648 ± 383 cells/mm^2^ for Egyptian^11^, 2732 ± 258 cells/mm^2^ for Thai^12^, 2732 ± 305 cells/mm^2^ for Turkish^13^, and 2932 ± 363 cells/mm^2^ for Chinese^14^ individuals. Although several studies have evaluated the status of ECD or endothelial morphology from various countries^10–16^, few have reported on healthy data collected from Japanese patients^17^. Furthermore, previous reports lack information on healthy patients aged < 40 years and none of the reports evaluated the healthy morphology. Because patients who use contact lens for refractive correction tend to be relatively young^18^, it is essential to determine the value and distribution of corneal endothelial density and morphology of healthy young patients for the accurate assessment of contact lens usage. Therefore, we aimed to analyze corneal ECD and morphology in healthy Japanese individuals without a history of contact lens usage, ophthalmic surgery, or ocular disease.

## Results

We included 16842 eyes of 8421 patients in the study (age, 19.6 ± 8.7 years; range, 6–70 years) (Table 1). A total of 6668 eyes of 3334 male patients (age, 19.3 ± 7.8 years) and 10,174 eyes of 5087 female patients (age, 19.8 ± 9.1 years) were included.

**Table 1:** Demographic data of ophthalmologically healthy Japanese eyes.

Sex	Side of the eye	N (eyes)	Age (years)	ECD (cells/mm^2^)	CV (%)	6A (%)	Cell area (μm^2^)
Total	Total	16842	19.6 ± 8.7	3109.0 ± 303.7	27.6 ± 5.1	65.9 ± 11.4	324.9 ± 32.5
	Right	8421	–	3117.0 ± 303.0	27.6 ± 5.1	66.0 ± 11.5	324.1 ± 32.4
	Left	8421	–	3101.8 ± 304.3	27.6 ± 5.1	65.9 ± 11.3	325.6 ± 32.7
Male	Total	6668	19.3 ± 7.8	3058.2 ± 292.9	27.1 ± 4.9	67.3 ± 11.2	330.2 ± 32.2
	Right	3334	–	3067.6 ± 293.4	27.1 ± 4.9	67.3 ± 11.3	329.3 ± 32.1
	Left	3334	–	3048.7 ± 292.2	27.1 ± 4.9	67.2 ± 11.1	331.1 ± 32.3
Female	Total	10174	19.8 ± 9.1	3142.9 ± 306.0	27.9 ± 5.1	65.1 ± 11.4	321.3 ± 32.2
	Right	5087	–	3149.3 ± 304.8	27.9 ± 5.1	65.1 ± 11.3	320.7 ± 32.1
	Left	5087	–	3136.6 ± 307.1	27.9 ± 5.1	65.0 ± 11.4	322.0 ± 32.4

ECD, corneal endothelial cell density; CV, coefficient of variation in cell area; 6A, appearance rate of hexagonal cells.

**Figure 1:**
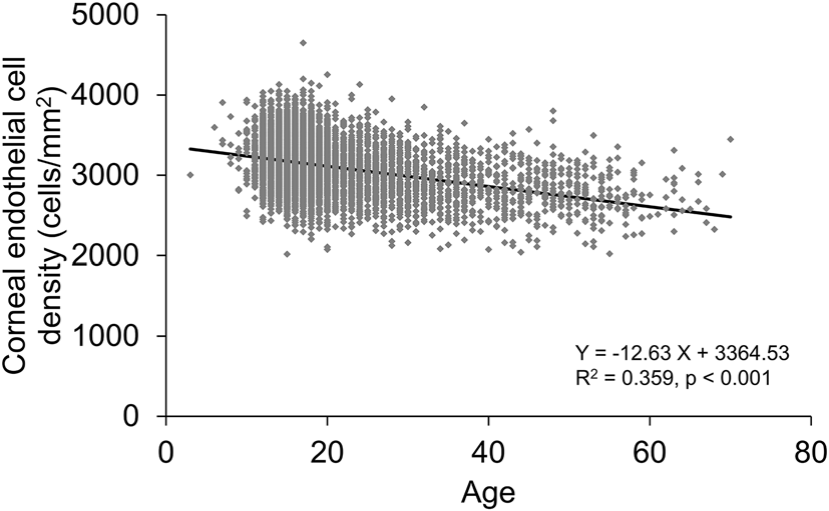
Association between corneal endothelial cell density and age in ophthalmologically healthy Japanese. Based on the linear regression model, a significant mild negative association between corneal endothelial cell density and age was observed (Y =  − 12.63 X + 3364.53, R^2^ = 0.359, p < 0.001).

**Figure 2:**
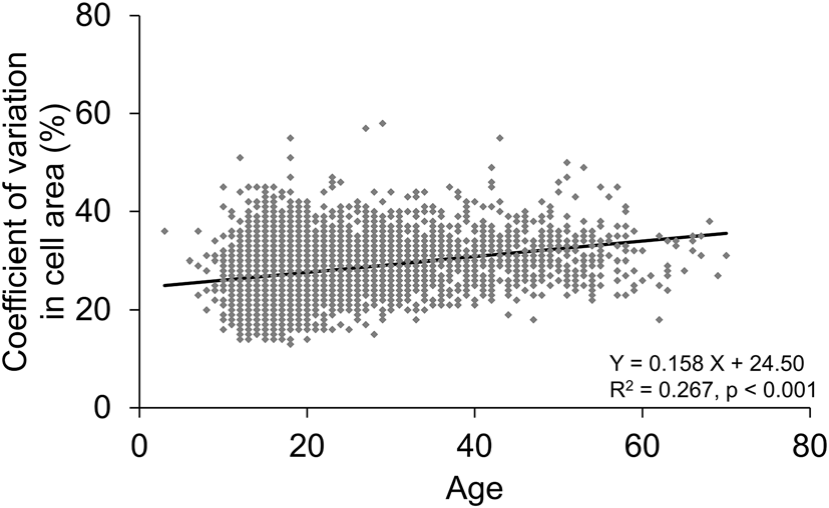
Association between the coefficient of variation in cell area and age in ophthalmologically healthy Japanese. Based on the linear regression model, a significant weak positive association between the coefficient of variation in cell area and age was observed (Y = 0.158 X + 24.50, R^2^ = 0.267, p < 0.001).

**Figure 3:**
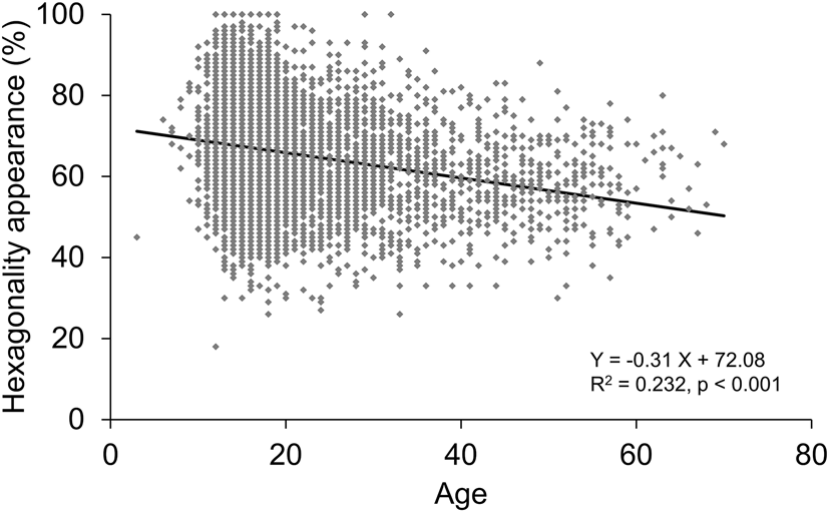
Association between the hexagonal appearance of corneal endothelial cell and age in ophthalmologically healthy Japanese. Based on the linear regression model, a significant weak negative association between the hexagonal appearance of corneal endothelial cell and age was observed (Y =  − 0.31 X + 72.08, R^2^ = 0.232, p < 0.001).

**Figure 4:**
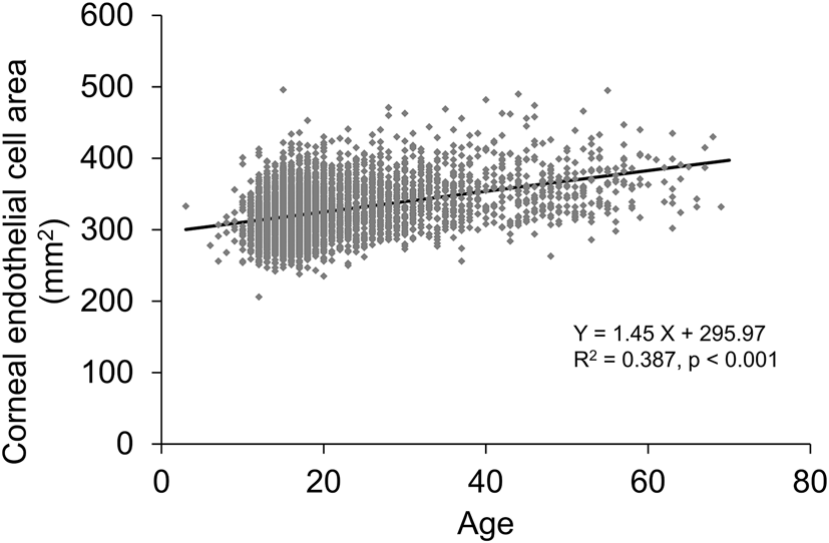
Association between corneal endothelial cell area and age in ophthalmologically healthy Japanese. Based on the linear regression model, a significant mild positive association between corneal endothelial cell area and age was observed (Y = 1.45 X + 295.97, R^2^ = 0.387, p < 0.001).

In the stratified data based on the age of 10 years, in the 1–10-year group, the mean ECD was the highest, mean the coefficient of variation (CV) was the lowest, mean appearance rate of hexagonal cells (6A) was the highest, and mean cell area was the lowest (Table 2). ECD was significantly negatively correlated with age (p < 0.001, R^2^ = 0.359, Fig. 1). ECD reduced to − 12.63 cells/mm^2^ per year, and the reduction rate was 0.42%/year in the total population. In the stratified age category, ECD had the greatest reduction in the 11–20-year group (− 19.3 cells/mm^2^/year), which was significantly correlated with age (p < 0.001). The CV in cell area was significantly positively correlated with age (p < 0.001, R^2^ = 0.267, Fig. 2) while the 6A was significantly negatively correlated with age (p < 0.001, R^2^ = 0.232, Fig. 3). The cell area was also significantly correlated with age (p < 0.001, R^2^ = 0.387, Fig. 4), gradually increasing with age. Correlations between age and ECD, CV, 6A, and cell area were confirmed with the data of all included eyes (bilaterally) as well as with those of unilateral eyes (all p < 0.001).

**Table 2:** Corneal endothelial cell density and morphology of ophthalmologically healthy Japanese eyes according to age category.

Age category	N (eyes)	Mean age (years)	ECD (cells/mm^2^)	CV (%)	6A (%)	Cell area (μm^2^)
1–10	96	9.3 ± 1.4	3314.5 ± 334.5	26.1 ± 5.3	69.8 ± 10.5	304.7 ± 33.9
11–20	12540	15.8 ± 2.2	3160.3 ± 284.5	26.9 ± 4.8	67.3 ± 11.2	319.2 ± 28.8
21–30	2608	24.8 ± 2.8	3027.6 ± 287.0	29.0 ± 4.9	62.9 ± 10.8	333.9 ± 32.5
31–40	828	34.7 ± 2.8	2874.2 ± 283.8	30.3 ± 5.2	60.2 ± 10.7	352.6 ± 35.8
41–50	478	45.1 ± 2.8	2814.4 ± 298.4	31.3 ± 5.3	59.2 ± 9.8	360.3 ± 39.5
51–60	246	54.3 ± 2.5	2734.5 ± 273.5	31.4 ± 5.3	58.7 ± 9.5	365.5 ± 37.2
61–70	46	64.5 ± 2.4	2740.0 ± 289.5	30.3 ± 4.2	61.3 ± 8.9	372.6 ± 35.9

ECD, corneal endothelial cell density; CV, coefficient of variation in cell area; 6A, appearance rate of hexagonal cells.

Multivariate linear regression analysis revealed that higher age and being male were significantly correlated with lower ECD (p < 0.001, Table 3). Higher age was also significantly related to higher CV, lower 6A, and larger cell area (all p < 0.001, Table 3). These correlations were confirmed with the data of all included eyes (bilaterally) as well as with those of unilateral eyes (all p < 0.001).

**Table 3:** Multiple linear regression analysis of endothelial cell density and morphology of healthy Japanese eyes.

		Unstandardized coefficient β	Partial correlation coefficient	P-value
ECD (cells/mm^2^)	Sex (male to female)	− 87.46	− 0.15	< 0.001
	Age (year)	− 12.76	− 0.37	< 0.001
CV (%)	Sex (male to female)	− 0.72	− 0.07	< 0.001
	Age (year)	0.16	0.27	< 0.001
6A (%)	Sex (male to female)	2.04	0.09	< 0.001
	Age (year)	− 0.31	− 0.23	< 0.001
Cell area (μm^2^)	Sex (male to female)	9.19	0.15	< 0.001
	Age (year)	1.46	0.39	< 0.001

ECD, corneal endothelial cell density; CV, coefficient of variation in cell area; 6A, appearance rate of hexagonal cells.

Regarding difference in sex, ECD and CV were significantly higher in female than male individuals after adjusting for age (p = 0.018 and 0.011, respectively; Table 4). 6A and cell area were significantly higher in male than female individuals (0.046 and < 0.001, respectively; Table 4). These correlations in ECD, CV, 6A, and cell area were confirmed with the data of all included eyes (bilaterally) as well as with those of unilateral eyes (p = 0.0053, < 0.001, < 0.001, and = 0.0237, respectively).

**Table 4:** Comparison of endothelial cell density and morphology of healthy Japanese eyes according to sex.

Sex	Mean age (years)	ECD (cells/mm^2^)	CV (%)	6A (%)	Cell area (μm^2^)
Male	19.3 ± 7.8	3058.2 ± 292.9	27.1 ± 4.9	67.3 ± 11.2	330.2 ± 32.2
Female	19.8 ± 9.1	3142.9 ± 306.0	27.9 ± 5.1	65.1 ± 11.4	321.3 ± 32.2
P*-value*	< 0.001	0.018	0.011	0.046	< 0.001

ECD, corneal endothelial cell density; CV, coefficient of variation in cell area; 6A, appearance rate of hexagonal cells.

## Discussion

We analyzed corneal ECD and morphology in 16842 eyes of young healthy Japanese patients, which is the largest number of the eyes compared to past reports, and demonstrated that the mean ECD was 3109.0 ± 303.7 cells/mm^2^ in healthy Japanese patients aged 19.6 ± 8.7 years (range, 6–70 years). Higa et al. previously reported that ECD was 2943 ± 387 cells/mm^2^ in ophthalmologically healthy Japanese patients aged more than 40 years, and the reduction was associated with age^17^. Our data were consistent with those of previous reports, including patients aged 40–50 years, from various countries as mentioned above, such as Nigeria (2610 ± 372)^10^, Egypt (2648 ± 383)^11^, Thailand (2732 ± 258)^12^, China (2932 ± 363)^14^, Turkey (2671 ± 356)^16^, the Philippines (2798 ± 307)^19^, and Malaysia (2648 ± 310)^20^. On the contrary, normal ECD data of young patients were scarce^21,22^. Thus, our study revealed significant information on corneal parameters in young patients aged less than 20 years and reinforced the previous studies conducted in Japan^17^. Furthermore, Shen et al. reported that race significantly affected ECD, and Matsuda et al. also reported that the averaged ECD is reportedly higher in Asian eyes than in Caucasian eyes; our results supported those of previous studies^23,24^.

The negative correlation between ECD and age that was clearly observed in our results was consistent with the results of previous studies^10–12,14,16,19,20,25^. The ECD reduction rate was 0.42%/year, close to that previously reported (0.3–0.5%)^8,14,26^. Corneal diameter was hypothesized to be associated with ECD difference among various populations^23^. However, we did not review the corneal diameter data because these were unavailable in all patients. Moreover, female patients had significantly higher ECD than male patients (Tables 3 and 4), which was consistent with the result of a previous study in Japan^17^. The reason for the difference between sexes is currently unknown. While some studies reported that females had higher ECD than males, other studies found that there was no difference between the sexes^11,20^. Regression analysis also revealed age-related changes, such as a decrease in 6A and increases in CV and cell area. These parameters indicate corneal endothelial stability in terms of cell morphology. Because corneal endothelial cells substantially decrease, when the cells die, the remaining cells will change their form and expand to cover the endothelial surface. Polymegathism, which is an increased variation in endothelial cell size, was rarely observed in young patients according to a previous report^15^. Additionally, our results demonstrated that corneal endothelium in female individuals had significantly higher ECD, higher CV, and lower 6A compared with those in male individuals, although low CV and high 6A are expected in patients with high ECD in general. This observational data was interesting, but the underlying reason is currently unknown based on the available data.

Age-related changes in ECD, CV, 6A, and cell area were suggested to be different among the groups based on the stratified data in Table 2. The result suggested that corneal endothelial cell distribution changed more acutely when patients were young and that the amount of morphological change would decrease with aging. These changes in corneal parameters could be related to the corneal diameter, as mentioned above^23^, considering that the cornea becomes larger as individuals age. This morphological change would require further analysis by conducting a longitudinal, observational study.

Soft contact lens usage is prevalent among young patients who desire to correct their myopia without glasses. Some users of soft contact lens experience corneal endothelial damage owing to mechanical stress or oxygen pressure reduction^6,27^. Therefore, it is critical to determine the normal range of ECD in young patients to understand ECD reduction and compare this reduction among individuals. However, surprisingly, the accumulated knowledge about ECD in young patients was insufficient, probably because young ophthalmologically healthy patients rarely visit hospitals. Furthermore, if they visit hospitals, ECD is not always evaluated. Therefore, we focused on ECD in healthy young patients and demonstrated normalized data in young patients without ophthalmologic disease. Because some corneal parameters are different among different populations, a future clinical study on corneal parameters including healthy young patients in various populations is required.

The current study has some limitations. First, the data included in the current study was obtained using three different types of corneal specular microscopes, which could affect the results. However, the specular microscopes used were basically similar with respect to the microscopic examination methodology. Contact specular microscopy is reportedly useful for further accurate evaluation, but a previous report demonstrated no difference when comparing the results of contact and noncontact specular microscopy^28^. Second, considering the retrospective nature of the study, some examination items were not available. Especially, high myopia was recently reported as correlated with CV and 6A of the corneal endothelium^29^; the refractive information is also important. To overcome these limitations, further prospective clinical studies with more detailed information of refractive error and history of systemic diseases, such as diabetic mellitus, that could affect the status of corneal endothelial cells should be conducted^30^.

In conclusion, we demonstrated normalized data of ECD and morphological parameters and changes per year in a large number of young healthy Japanese patients. It is important to monitor corneal endothelial cells in young patients to assess the possible risk of endothelial damage and to avoid future complications.

## Methods

This retrospective observational study was approved by the Institutional Review Board of Miyata Eye Hospital (Miyazaki, Japan) and adhered to the tenets of the Declaration of Helsinki. Informed consent was obtained from all the person or guardian by an opt-out procedure.

We included eyes of Japanese patients who did not have ophthalmologic diseases, other than refractive errors, and whose corneal endothelia were evaluated before the initiation of contact lens usage at Miyata Eye Hospital from January 1996 to December 2015. We excluded eyes that had previous history of ophthalmologic diseases or contact lens usage. Additionally, we excluded any patients whose cornea was evaluated only unilaterally. We retrospectively reviewed patients’ age, ECD, and morphological data (e.g., 6A, CV and cell area) obtained from their medical charts.

Data of corneal endothelium were obtained using noncontact specular microscopy (FA-3509, SP-8000, and CA-2308, Konan, Nishinomiya, Japan). An endothelial cell image around the center of the cornea was captured, and all endothelial cells within the captured image of a 0.24 × 0.4-mm area were automatically traced. Moreover, the ECD (cell/ mm^2^) of the central area of the cornea was automatically calculated. The process was commonly performed in all noncontact specular microscopy techniques. Simultaneously, 6A, CV, and cell area were also evaluated from the image owing to the program set in the noncontact specular microscope. For the statistical analysis regarding the association between age and corneal ECD or morphology, we selected only the right eye from each patient. The included patients were categorized into groups of allocation by stratifying age in a 10-year interval.

For statistical analysis, linear regression model was applied to assess the association between age and ECD, 6A, CV, or cell area. Stepwise multivariate linear regression analyses were performed to determine the correlations among ECD, sex, and age. Analysis of covariance was performed to compare ECD, 6A, CV, or cell area to adjust for the covariates of age. Statistical analyses were performed using the BellCurve for Excel (Social Survey Research Information, Tokyo, Japan) and GraphPad Prism 9 (GraphPad Software Inc., CA, USA). All data are expressed as mean ± standard deviation unless otherwise mentioned. Two-tailed P-value < 0.05 was considered statistically significant.

## Data Availability

The data that support the findings of this study are available upon request from the corresponding author. The data are not publicly available because they contain information that could compromise the privacy of the research participants.
